# Omics Multi-Layers Networks Provide Novel Mechanistic and Functional Insights Into Fat Storage and Lipid Metabolism in Poultry

**DOI:** 10.3389/fgene.2021.646297

**Published:** 2021-07-07

**Authors:** Farzad Ghafouri, Abolfazl Bahrami, Mostafa Sadeghi, Seyed Reza Miraei-Ashtiani, Maryam Bakherad, Herman W. Barkema, Samantha Larose

**Affiliations:** ^1^Department of Animal Science, College of Agriculture and Natural Resources, University of Tehran, Karaj, Iran; ^2^Nuclear Agriculture Research School, Nuclear Science and Technology Research Institute, Karaj, Iran; ^3^Department of Cell and Molecular Biology, Faculty of Biological Sciences, Kharazmi University, Tehran, Iran; ^4^Department of Production Animal Health, University of Calgary, Calgary, AB, Canada; ^5^One Health at UCalgary, University of Calgary, Calgary, AB, Canada

**Keywords:** lipid metabolism, transcriptome, systems biology, interactive bipartite network, omics multilayer

## Abstract

Fatty acid metabolism in poultry has a major impact on production and disease resistance traits. According to the high rate of interactions between lipid metabolism and its regulating properties, a holistic approach is necessary. To study omics multilayers of adipose tissue and identification of genes and miRNAs involved in fat metabolism, storage and endocrine signaling pathways in two groups of broiler chickens with high and low abdominal fat, as well as high-throughput techniques, were used. The gene–miRNA interacting bipartite and metabolic-signaling networks were reconstructed using their interactions. In the analysis of microarray and RNA-Seq data, 1,835 genes were detected by comparing the identified genes with significant expression differences (p.adjust < 0.01, fold change ≥ 2 and ≤ −2). Then, by comparing between different data sets, 34 genes and 19 miRNAs were detected as common and main nodes. A literature mining approach was used, and seven genes were identified and added to the common gene set. Module finding revealed three important and functional modules, which were involved in the peroxisome proliferator-activated receptor (PPAR) signaling pathway, biosynthesis of unsaturated fatty acids, Alzheimer’s disease metabolic pathway, adipocytokine, insulin, PI3K–Akt, mTOR, and AMPK signaling pathway. This approach revealed a new insight to better understand the biological processes associated with adipose tissue.

## Introduction

Total carcass fat of broilers varies depending on sex, poultry age, nutrition, and genetic factors (about 12%) (Sakomura et al., 2005). The predominant fats stored in a broiler carcass include two kinds of subcutaneous fat and ventricular fat (approximately 18 to 22% of carcass fat) stored in the ventricular area (Crespo and Esteve- Garcia, 2001). For humans, as the foremost consumer of poultry meat, over-fat storage in skeletal muscle is associated with metabolic diseases such as type 2 diabetes and cardiovascular disease and subsequently will lead to the risk of a heart attack. Fat production in poultry is a high-inheritance polygenic trait regulated by various behavioral, environmental, and hormonal factors (Le Mignon et al., 2009).

Many studies have identified genes related to storage lipids in broilers (Lagarrigue et al., 2006; Pinto et al., 2010; Nones et al., 2012). On the other hand, the integration of high-throughput genomic DNA and RNA sequencing leads to the identification of genomic regions that control traits at the whole genome scale (Cesar et al., 2018). Some studies of two poultry groups, obese [high fat (HF)] and lean [low fat (LF)], indicated genes associated with lipogenic pathways (Ji et al., 2012, 2014). By comparing expressed genes, numerous identified genes were related to endocrine, hemostatic, lipolytic, and lipid transduction (Resnyk et al., 2013).

In addition to identifying genes and pathways associated with lipid metabolism, a holistic approach for gene expression should be examined. MicroRNAs are regulatory molecules with a length of 19–25 nucleotides (Bartel, 2004). Mature microRNAs lead to decomposition or inhibit translation by complete or partial coupling to target mRNAs (usually paired with the 3′UTR region) (Iorio et al., 2011). We have witnessed the emergence of various areas in biology. One of these areas is the application of bioinformatics and systems biology and integrated multi-omics data. In major systems biology, researchers have attempted to identify the cellular system, formulate cell behaviors, and then design a cell model by combining genomic, transcriptomic, proteomic, and metabolomic layers (Cole et al., 2013). In this regard, interactive bi-partite networks of gene–miRNA are used in several studies to discover functional modules (Huang et al., 2006; Bahrami et al., 2017a,b).

However, identification of upstream and downstream genes, reconstruction of networks, bipartite interaction network of gene–miRNA, and metabolic-signaling networks involved in metabolism and adipose storage (particularly abdominal fat using high-throughput data in broilers) have not been reported. Fat storage in broilers is considered to be an important economic trait concerning high growth rate. Based on previous studies of fat metabolism in the body and signaling pathways related to fat storage and transmission in laboratory species, it was assumed that the two broiler groups of high-abdominal fat and low-abdominal fat have gene expression differences in metabolism and fat storage.

Accordingly, this study aims to use an integration of RNA-Seq and microarray data approach to identify and classify candidate genes and miRNAs involved in lipolysis and lipogenesis. In addition to the comprehensive survey of lipid metabolism, this study will focus on (1) reconstruction of the interactive bi-partite network of gene–miRNA (bi-partite networks are a particular class of complex networks, whose nodes are divided into two sets of genes and miRNA), (2) identification of functionally relevant modules (each of a set of genes or independent genes that can be used to construct a more complex structure), and (3) reconstruction of the metabolic-signaling network associated with the process of metabolism and fat storage in broilers.

## Materials and Methods

**FIGURE 1:**
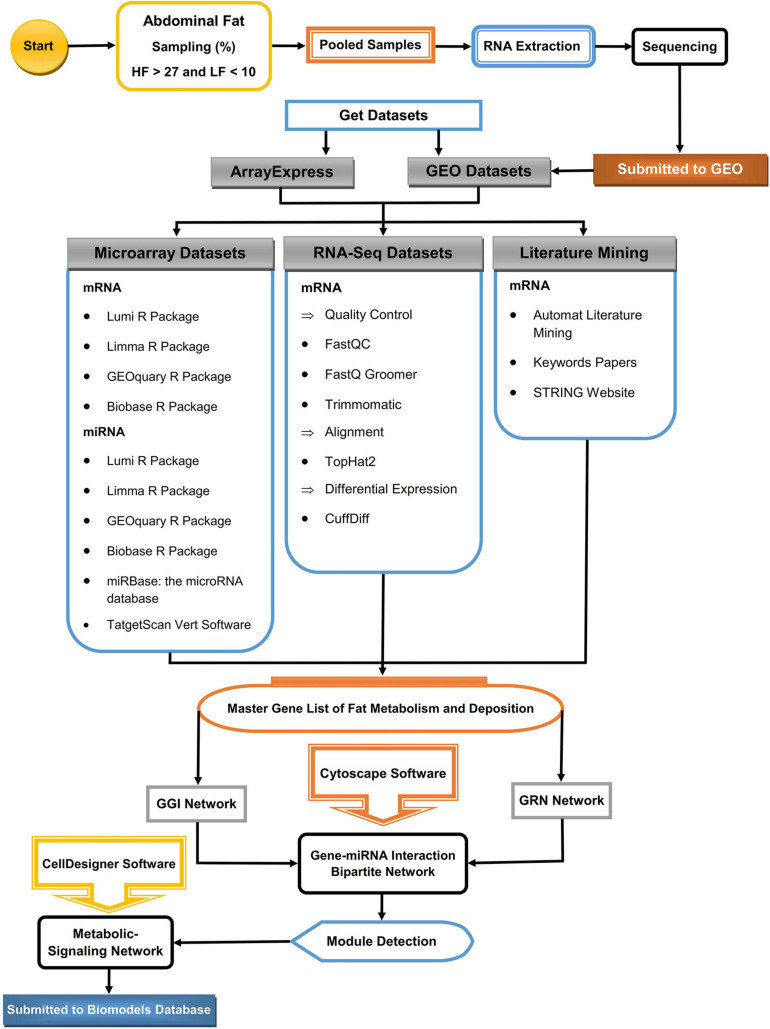
Schematic for analysis view of the workflow to reconstruct the metabolic pathways of abdominal fat storage in poultry. The main gene list was prepared from three RNA-Seq and microarray data sets. The Gene–Gene Interaction Network (GGI), Gene Regulatory Network (GRN), and interactive bi-partite network of gene–miRNA network were reconstructed using Cytoscape. Functional modules were detected using related plugin in Cytoscape and the metabolic-signaling network using CellDesigner.

Figure 1 and Supplementary Table 1 show the simple overall workflow for analyzing and finding functionally relevant modules with HF and LF storage in the broilers.

### Poultry’s Tissue Preparation

The 18 chickens used in this study were divergently selected based on the amount of carcass fat percentage at 42 days of age (slaughtering time). Chickens were bred and raised at the animal farm of Tehran University, Iran. Nine chickens were in the HF group (>27% fat storage) and nine chickens in the LF group (<10% fat storage). Each group was divided into three subgroups with three chickens in each group. To eliminate other environmental effects and sampling error, abdominal adipose tissue samples of three chickens in each subgroup were pooled. Therefore, we had three samples for HF and three samples for LF chickens, separately. In this regard, both groups were placed together and raised in floor pens (4.4 m × 3.9 m). Abdominal adipose tissue samples were immediately pooled (before RNA extraction), snap-frozen in liquid nitrogen, and stored at −80° C until further processing for RNA analysis.

### RNA Extraction

Abdominal fat aliquots from six chickens (three HF and three LF per age at 42 weeks) were homogenized, and total cellular RNA was extracted using guanidine thiocyanate and CsCl gradient purification, followed by DNase I treatment. The quality of RNA was determined with an RNA 6000 Nano Assay kit and the Model 2100 Bioanalyzer (Agilent Technologies, Palo Alto, CA, United States). All samples used for RNA analyses had an RNA integrity number (RIN) greater than 9.0.

### MiRNA-Seq Library Preparation and Sequencing

About 1 μg of total RNA from each sample was used to construct a small RNA library using the TruSeq Small RNA Sample Preparation kit (Illumina, San Diego, CA, United States). The kit was used according to the manufacturer’s instructions, which included ligating adapters to 3′ and 5′ end of the RNA molecules, reversely transcribing and amplifying libraries, purifying cDNA, and checking and normalizing libraries. All libraries were sequenced at Génome Québec (Montréal, Canada) using the HiSeq 2000 system (Illumina, San Diego, CA, United States) to generate 50-bp single reads.

### Data Mining

In the biological system and the reconstruction of biological networks, namely, gene regulation, interactions, protein–protein interaction (PPI), and metabolic networks, the first step is to collect and evaluate the available data. In this regard, data from this study were obtained by investigating and reviewing related articles and collecting microarray and RNA-Seq data from different databases, by searching the Gene Expression Omnibus (GEO) database^1^ and ArrayExpress^2^ for abdominal fat in various species, particularly for *Gallus gallus domesticus*. The accession numbers for the RNA-Seq and microarray data sets are presented in Table 1.

**TABLE 1:** GEO accession numbers for RNA-Seq and microarray data sets.

No.	No. sample(s)	GSE	Platforms	Data type	Contributor(s)
1	16	GSE49121	GPL16133 (Illumina HiSeq 2000)	RNA-Seq	Resnyk et al., 2017
2	24	GSE42980	GPL16133 (Illumina HiSeq 2000)	RNA-Seq	Resnyk et al., 2015
3	24	GSE37585	GPL1731 (DEL-MAR 14K Integrated Systems)	Microarray	Resnyk et al., 2013
4	24	GSE8812	GPL1731 (DEL-MAR 14K Integrated Systems)	Microarray	Resnyk et al., 2015
5	24	GSE45825	GPL1731 (DEL-MAR 14K Integrated Systems)	Microarray	Resnyk et al., 2017
6	8	GSE10052	GPL1731 (DEL-MAR 14K Integrated Systems)	Microarray	Byerly et al., 2010
7	28	GSE3867	GPL3265 (Chicken cDNA DDMET 1700 array version 1.0)	Microarray	Bourneuf et al., 2006

*GEO, gene expression omnibus.*

### Analysis of Microarray Data

Microarray data were pre-processed in software R, using package Lumi (Du et al., 2008) and Affy (Gautier et al., 2004). The processed data were then evaluated using packages Limma (Ritchie et al., 2015), GEOquary (Davis and Meltzer, 2007), and Biobase (Huber et al., 2015) (versions and parameters were used for analysis of microarray and RNA-Seq presented in Supplementary Table 1). Among the number of identified genes, the genes that were common in terms of five accession numbers (related to microarray data sets) were identified; and the gene list was considered as gene set 1 (Supplementary Table 2).

### RNA-Seq Data and Statistical Analyses

Various programs were used to analyze the RNA-Seq data related to the accession numbers. First, FastQC quality control software (Andrews, 2010) was used to control the quality of existing data. Sequences were trimmed for quality using Trimmomatic software (Bolger et al., 2014). Boxplot graphing of pre- and post-trimming reads confirmed the absence of outlier samples based on read count. After trimming, reads were mapped to the chicken genome assembly GRCg6a^3^ using Tophat (version 1.3.3) (Kim et al., 2013), followed by assembly and quantitation using CuffDiff software (v2.2.1.6) (Trapnell et al., 2010). The fragments per kilobase of exon per million fragments mapped (FPKM) threshold for detection of a gene was set at FPKM > 0.5. The resulting gtf files differential expression was assessed using Cuffdiff. The two-sided *p*-value was corrected using the false discovery rate (FDR), which accounts for multiple testing procedures. Genes with an FDR-adjusted *p*-value (*p* ≤ 0.05) and fold change ≥ 2 or ≤ −2 were considered to be differentially expressed (DE) transcripts. The genes that were common in terms of accession numbers (related to RNA-Seq data sets) were identified and considered as gene set 2 (Supplementary Table 3).

### Functional Gene Set Annotation and Enrichment

Gene ontology (GO) analysis, canonical pathway, and network identification were performed using Database for Annotation, Visualization and Integrated Discovery (DAVID)^4^, Bioinformatics Resources 6.8 with (Huang et al., 2009) the Kyoto Encyclopedia of Genes and Genomes (KEGG) database, g: profiler^5^ (Raudvere et al., 2019), GeneCards^6^, and PANTHER (Protein ANalysis THrough Evolutionary Relationships) (Mi et al., 2012).

### Main Gene List

Genes with significant differences related to microarray and RNA-Seq data were examined and listed as gene set 1 and 2, respectively. Finally, genes that were common in these two gene sets were chosen as the main gene list.

### Identification of miRNAs and Target Genes

Accession number GSE122224, which is related to miRNA in chicken and associated with lipid metabolism, was analyzed. The potentially targeted genes were predicted using miRWalk 3.0 (Sticht et al., 2018). The platform integrates information from different miRNA-target databases, including validated information and prediction data sets: MiRWalk (Dweep et al., 2014), miRDB^7^, miRMap (Vejnar and Zdobnov, 2012), miRNAMap (Hsu et al., 2008), miRanda^8^, miRBridge (Tsang et al., 2010), PICTAR2^9^, Targetscan (Grimson et al., 2007), PITA^10^, and RNA22 (Loher and Rigoutsos, 2012). The target genes that were predicted by at least five mentioned tools were chosen and submitted to DAVID, KEGG (the potential KEGG), Reactome pathways, and PANTHER databases for the enrichment target genes of each miRNA.

### Reconstruction of Omics Multilayered Networks

The miRNA–gene network was reconstructed based on the candidate genes, and the molecular interactions were documented in related papers and online interaction databases. PPI data were abstracted from the Biomolecular Interaction Network Database (BIND^11^), Database of Interacting Proteins (DIP^12^), Biological General Repository for Interaction Datasets (BioGRID^13^), and Protein–Protein Interactions Database (MIPS^14^). In addition, pathway data were obtained from searches in pathway databases, such as STRING^15^ (Szklarczyk et al., 2018) and GeneMania databases^16^ (Warde-Farley et al., 2010). Each gene and miRNA was entered into the database, and resulting interactions were imported to the networks using Cytoscape 3.7.2 (National Institute of General Medical Sciences, Bethesda Softworks, Rockville, MD, United States) (Shannon et al., 2003). Genes and miRNAs in generated networks are represented as nodes, and the interactions between these nodes as edges. The metabolic-signaling pathways involved in the lipid metabolism and storage were reconstructed by different databases and Cell Designer version 4.4.2 (Funahashi et al., 2008).

### Modules and Hub Node Detection

For finding sub-graphs and hub nodes (nodes with a high connectedness coefficient), MCODE, one of the Cytoscape plugins, was used. MCODE finds clusters (highly interconnected regions) in a network. Clusters mean different things in different types of networks. For instance, clusters in a PPI network are often protein complexes and parts of pathways, while clusters in a protein similarity network represent protein families (Bader and Hogue, 2003). MCODE effectively finds densely connected regions of a molecular interaction network, many of which correspond to known molecular complexes, based solely on connectivity data. Given that this approach to analyzing protein interaction networks performs well using minimal qualitative information implies that large amounts of available knowledge are buried in large protein interaction networks. More accurate data mining algorithms and systems models could be constructed to understand and predict interactions, complexes, and pathways by taking into account more existing biological knowledge. Structured molecular interaction data resources such as BIND will be vital in creating these resources (Bader et al., 2003).

## Results

Differentially expressed genes were defined as those having a significant adjusted *p*-value (<0.01), fold change (≥2, ≤−2), and FDR (≤0.05). Statistical analysis of the time-course microarray studies provided 1,451 significant genes from five data sets: the first data set (GSE37585: 612 DE genes), the second data set (GSE8812: 107 DE genes), the third data set (GSE45825: 582 DE genes), the fourth data set (GSE10052: 104 DE genes), and the fifth data set (GSE3867: 46 DE genes). In the data analysis of RNA-Seq, 1,867 genes were identified; and then 314 and 70 genes were detected after considering the threshold (p.adjust < 0.01 and fold change > 2) of expression change in accession numbers GSE49121 and GSE42980, respectively.

### Identification of miRNAs

Overall, 34 miRNAs were identified in data analysis of microRNAs differential expression, of which 19 upregulated miRNA and 15 downregulated genes were detected by considering the threshold (LogFC < −2, LogFC > 2, and p.adjust < 0.01) for DE in the deposited accession number (GSE122224) (Table 2).

**TABLE 2:** Genes and miRNAs, annotation, and genes involved in lipogenesis and lipolysis.

Lipogenesis					Lipolysis				
Gene	Gene expression		MiRNA expression		Gene	Gene expression		MiRNA expression	
	Downregulation	Upregulation	Downregulation	Upregulation		Downregulation	Upregulation	Downregulation	Upregulation
*THBS1*	*		−	gga-miR-6554-5p gga-miR-6667-5p gga-miR-6562-3p	*COLEC12*	*		−	gga-miR-6554-5p gga-miR-6554-3p gga-miR-6667-5p gga-miR-3532-5p gga-miR-466
*ANXA7*	*		−	gga-miR-466	*RGS19*	*		−	−
*TMEM258*		*	−	−	*HTR7L*		*	−	−
*DHCR7*		*	−	−	*G6PC*		*	−	−
*FADS2*		*	−	−	*HMGCR*		*	gga-miR-1710	−
*FASN*		*	−	−	*ACAT1*	*		−	−
*INSIG2*		*	gga-miR-7444-5p	−	*ADH1C*	*		−	−
*LCAT*		*	−	−	*APP*	*		−	gga-miR-6554-5p
*MVD*		*	−	−	*EHHADH*	*		−	−
*SCD*		*	−	−	*GAMT*	*		−	−
*SREBF1*		*	−	−	*HADHB*	*		−	−
*APOA1*	*		−	−	*HSD17B4*	*		−	−
*BCO2*	*		−	−	*HSD17B6*	*		−	−
*CYP27A1*	*		−	−	*IRS1*	*		−	gga-miR-6554-5p gga-miR-6562-3p gga-miR-466
*CYP2E1*	*		−	−	*PHYH*	*		−	−
*SLC2A2*	*		−	−	*SOD3*	*		−	−
					*TP53*	*		−	−
					*UCP3*	*		−	−

Upregulated and downregulated abdominal fat in genetically fat compared with lean chickens. *Gene or miRNA was up-/downregulated in the biological processes.

### Identification of Common Genes Available in Gene Sets 1 and 2

Thirty-four genes were common in two gene sets 1 and 2 relating to microarray and RNA-Seq data sets, respectively. In this regard, 16 and 18 genes were associated with lipogenesis and lipolysis processes, respectively (Table 2). *THBS1* and *INSIG2* genes in the gene set were associated with the lipogenesis process; and *COLEC12*, *HMGCR*, *APP*, and *IRS1* genes were associated with the lipolysis process, which was closely suppressed by miRNAs (Table 2).

### Main Gene List

Literature related to lipid metabolism was also reviewed to increase study accuracy and seven genes. If the genes did not exist in the list of evaluated data sets, they were selected and added to the gene list. The selected seven genes included *BACE1*, *BACE2*, *PSEN1*, *PSEN2*, *PERP*, *SIK1*, and *LOC421682* genes. The list of genes in Table 2 (41 genes) was named as the main gene list or reference genes (Supplementary Table 4).

### Gene–Gene Interaction Network, Gene Ontology Terms, and Pathways

**FIGURE 2:**
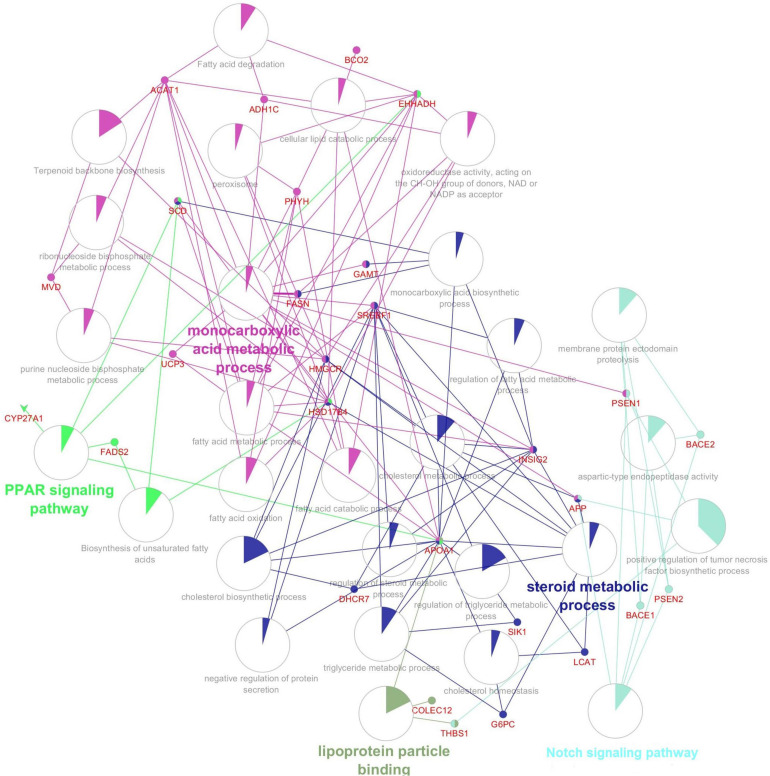
The gene, gene ontology and pathway, and related interaction network involved in the abdominal fat storage of the poultry.

Figure 2 shows the network of the reconstruction of gene–gene interactions (gene–gene interaction (epistasis) is the effect of one gene on a disease or traits modified by another gene or several other genes), GO (describes our knowledge of the biological domain with respect to three aspects: Molecular Function, Cellular Component, and Biological Process), terms, and pathways. In this network, *APP*, *SREBF1*, *HMGCR*, *FADS2*, *SCD*, *ACAT1*, *FASN*, *HADHB*, and *EHHADH* genes had the highest interaction (connectedness) with other genes in the network.

### Reconstruction of the Interactive Gene–miRNA Bipartite Network

**FIGURE 3:**
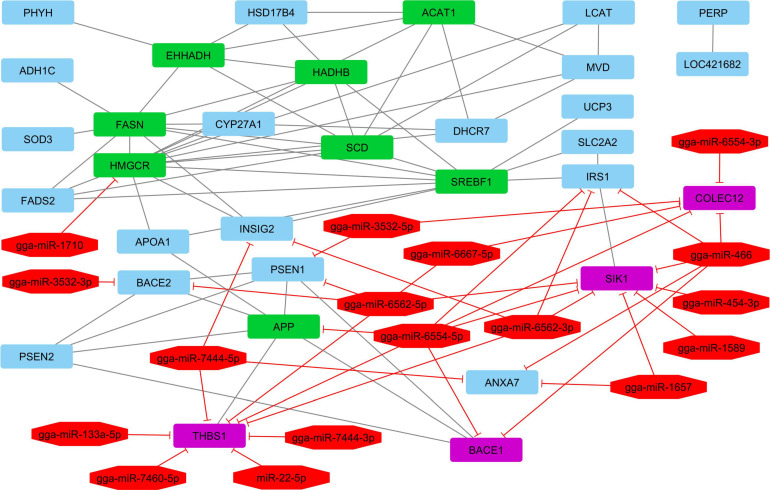
Interactive bipartite network (gene–miRNA) affecting the abdominal fat storage and metabolism in the poultry. In this network, the quadrilateral points represent genes, and the octagonal points represent miRNAs. About miRNAs and target genes, the edges indicate the suppressing role of miRNAs. The edges also represent the gene–gene interactions. The green quadrilateral nodes represent the hub genes. Purple quadrilateral nodes are the genes with the highest suppression by miRNAs.

The network contains 49 nodes (including 32 genes and 17 miRNAs) and 95 edges. The reconstructed network with.cys format was stored for further analyses (Figure 3).

### Important and Functional Network Modules or Sub-Networks

**FIGURE 4:**
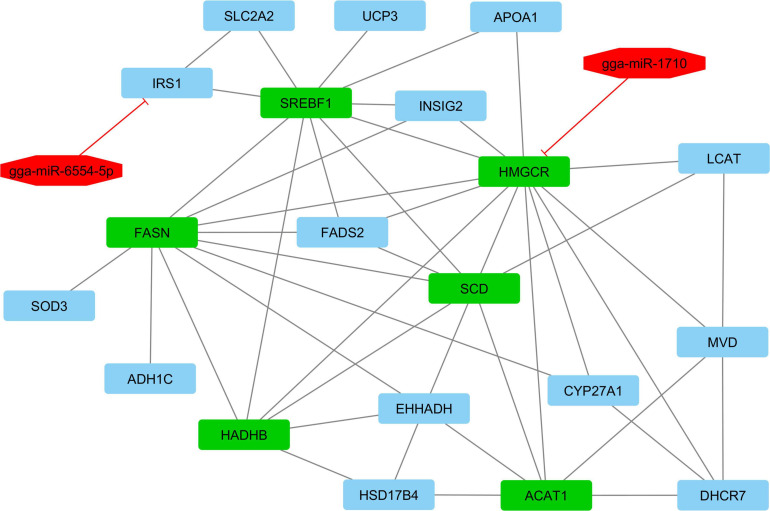
Module 1: 20 genes and two miRNAs in the interactive bipartite network of gene–miRNA. In this network, the quadrilateral points represent the genes, and the octagonal points represent miRNAs. In this interactive bipartite network, the gene–miRNAs of quadrilateral nodes represent genes and octagonal nodes represent miRNAs. For miRNAs and target genes, the edges indicate the suppressing roles of miRNAs. The edges of genes also indicate the gene–gene interactions. The green quadrilateral nodes represent the hub genes. Purple quadrilateral nodes have the highest rates of suppression by miRNAs.

**FIGURE 5:**
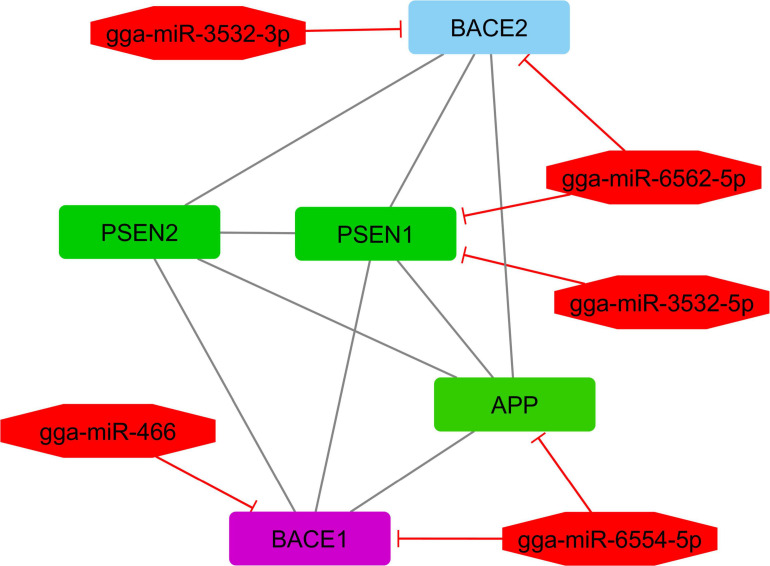
Module 2: five genes and five miRNAs in the interactive bipartite network of gene–miRNA. In this network, the quadrilateral points represent genes; and the octagonal points represent miRNAs. In this interactive bipartite network of gene–miRNA, quadrilateral nodes represent genes; and octagonal nodes represent the miRNAs. For miRNAs and target genes, the edges indicate the suppressing roles of miRNAs. The edges of genes also indicate the gene–gene interactions. The green quadrilateral nodes represent the genes with the highest gene–gene interactions with other genes in the network (or hub genes). Purple quadrilateral nodes indicate the genes with the highest suppression by miRNAs.

**FIGURE 6:**
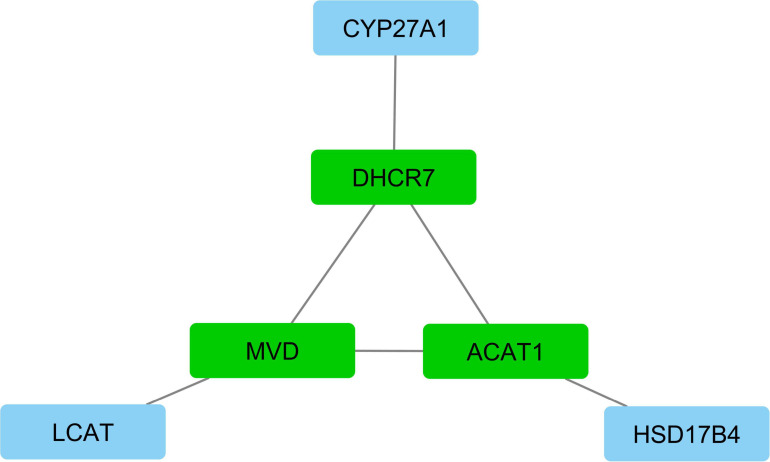
Module 3: six genes in interactive bipartite network of gene–miRNA. In this network, the quadrilateral nodes represent genes; and edges indicate the gene–gene interaction effects. Green quadrilateral nodes represent the hub genes in the network. Blue nodes represent other genes in the network.

According to interactive gene–miRNA bipartite network and sub-networks or module finding analysis, three modules were identified. These modules contained 31 genes and seven miRNA as presented in Table 3. The table also presents important signaling pathways and cellular processes (metabolic pathways) (Figure 2). Module 1 contains 22 nodes (20 genes and two miRNAs) and 47 edges (Figure 4). Module 2 contains 10 nodes (five genes and five miRNAs) and 16 edges (Figure 5); and Module 3 includes six nodes (six genes) and six edges (Figure 6).

**TABLE 3:** Main, modules, genes, miRNAs, signaling pathway, and phenotypic explanations in the integrated gene–miRNA bipartite network involved in fat metabolism and deposition.

	Genes		miRNAs			
Module	Downregulated	Upregulated	Downregulated	Upregulated	Signaling pathway	Explanation
Main	COLEC12, ANXA7, SOD3, SIK1, UCP3, ADH1C, SLC2A2, IRS1, BACE2, PERP, LOC421682, PHYH, CYP27A1, HADHB, THBS1, APOA1, BACE1, ACAT1, HSD17B4, EHHADH, APP, PSEN2, PSEN1	LCAT, INSIG2, FADS2, SCD, DHCR7, FASN, SREBF1, MVD, HMGCR	gga-miR-454-3p, gga-miR-7460-5p, gga-miR-133a-5p, gga-miR-1710, gga-miR-1589, gga-miR-22-5p, gga-miR-7444-3p, gga-miR-1657, gga-miR-7444-5p	gga-miR-6562-3p, gga-miR-3532-5p, gga-miR-6667-5p, gga-miR-6554-3p, gga-miR-6562-5p, gga-miR-3532-3p, gga-miR-6554-5p, gga-miR-466	PPAR/AMPK	Fatty acid metabolism Fatty acid degradation Terpenoid backbone biosynthesis Biosynthesis of unsaturated fatty acids Metabolic pathways Alzheimer disease
1	UCP3, SLC2A2, APOA1, IRS1, SOD3, ADH1C, HADHB, EHHADH, CYP27A1, HSD17B4, ACAT1	SREBF1, INSIG2, HMGCR, LCAT, FADS2, FASN, SCD, MVD, DHCR7	gga-miR-1710	gga-miR-6554-5p	PPAR/AMPK	Fatty acid metabolism Fatty acid degradation Terpenoid backbone biosynthesis Biosynthesis of unsaturated fatty acids Metabolic pathways Valine, leucine and isoleucine degradation Cholesterol metabolism Primary bile acid biosynthesis
2	BACE2, PSEN2, PSEN1, APP, BACE1	−	−	gga-miR-3532-3p, gga-miR-3532-5p, gga-miR-466, gga-miR-6554-5p, gga-miR-6562-5p	Notch signaling pathway	Alzheimer disease
3	CYP27A1, ACAT1, HSD17B4	DHCR7, MVD, LCAT	−	−	−	Primary bile acid biosynthesis Terpenoid backbone biosynthesis Metabolic pathways Cholesterol metabolism Fatty acid metabolism

### Reconstruction of the Metabolic-Signaling Network

**FIGURE 7:**
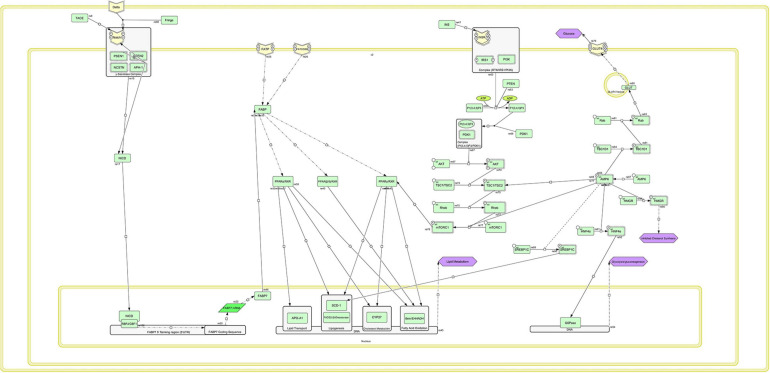
Schematic of the regenerated metabolic-signaling network associated with fat metabolism and storage using CellDesigner.

Based on pathway analysis, the crucial pathways were identified and reconstructed. For this purpose, the gene lists were first input into DAVID and STRING to identify biological processes, the involvement of cellular components, molecular functions, and KEGG pathways that were significantly different between two lines (to identify metabolic pathways and signaling). Different genes express identified Pathways such as Notch signaling pathways relating to Alzheimer’s disease, peroxisome proliferator-activated receptor (PPAR), adipocytokine, insulin, PI3K–Akt, mTOR, and AMPK signaling pathways. Finally, resources were reviewed for each of the identified paths, using different databases and Cell Designer software version 4.4.2; the reconstruction is illustrated in Figure 7.

## Discussion

The prioritization of abdominal fat tissue in broiler chickens to identify genes involved in metabolism and fat storage is due to the fact that it can be as a proxy model in other species and individuals of a species due to its specific metabolic characteristics (Resnyk et al., 2015). The present study integrated different data sets in distinguished conditions to identify the most important genes involved in lipid metabolism. As a result, we detected a total of 34 common genes that played roles in the main process of synthesis route control, metabolism and fat storage, and signaling pathways of endocrine glands activated by adipokines, AMPK, and PPAR.

The lower expression of a large number of genes associated with the lipolysis indicated a reduction in decomposition of fats and then an increase in the anabolism and fat storage in broiler chickens, especially in abdominal fat tissue. On the contrary, the higher expression of a large number of genes in the gene set associated with the lipogenesis confirms the increase in metabolism and abdominal fat storage.

In most similar studies published on different species, it has been concluded that multi-omics data sets or omics multilayered networks provide a valuable resource for comparative analyses with other experimental data sets. Also, applications for data integration and analysis can be demonstrated and provide novel functional insights (Yao et al., 2015; Suravajhala et al., 2016; Hasin et al., 2017; Arora et al., 2018; Backman et al., 2019; Dao et al., 2019; Corral-Jara et al., 2020; Lee et al., 2020). In one study, an attempt has been made to investigate the effects of a transgenic supplement in mice using a molecular systems biology approach and a combination of statistical tools using high-throughput techniques. They concluded that the integration of omics data provides better molecular insight into the relationships between biological variables. Thus, such approaches can be effective in detecting mechanical, molecular, and biochemical interactions (Zhang et al., 2019).

Chickens with greater abdominal fat had hyperplasia and hypertrophy of fat cells at younger ages compared with chickens with lower abdominal fat. *SREBF1*, *SREBF2*, *SCD*, and *FASN* were among the most important genes that play major roles in fat storage and metabolism (Resnyk et al., 2013). *THBS1*, *ANXA7*, *APOA1*, *BCO2*, *CYP27A1*, *CYP2E1*, and *SLC2A2* genes were downregulated, whereas *TMEM258*, *DHCR7*, *FADS2*, *FASN*, *INSIG2*, *LCAT*, *MVD*, *SCD*, and *SREBF1* genes were upregulated in the lipogenesis process. Additionally, *COLEC12*, *RGS19*, *ACAT1*, *ADH1C*, *APP*, *EHHADH*, *GAMT*, *HADHB*, *HSD17B4*, *HSD17B6*, *IRS1*, *PHYH*, *SOD3*, *TP53*, and *UCP3* were downregulated, whereas *HTR7L*, *G6PC*, and *HMGCR* genes were upregulated in the lipolysis process. Briefly, hub genes in this study were *APP*, *SREBF1*, *HMGCR*, *FADS2*, *SCD*, *ACAT1*, *FASN*, *HADHB*, and *EHHADH* (Figure 2).

The *APP* gene was downregulated in the lipolysis process because the *APP* gene is a cell surface receptor and an extra-membrane precursor protein that is decomposed by enzymes to form a number of peptides. Some of these peptides are secreted and can be bound to an acetyl transferase complex, APBB1/TIP60, to strengthen the transcription activities, while other proteins create amyloid plaques in brains of patients with Alzheimer’s disease (Almkvist et al., 2019). It enhances the transcription through binding to APBB1/KAT5 and inhibits Notch signals through interaction with Numb.

*Sterol regulatory element-binding transcription factor 1 (SREBF1)* gene was upregulated in the lipogenesis process because the *SREBF1* is a protein-encoding gene. Fatty liver disease is a *SREBF1* gene-related disease; and the mTOR signaling pathway is a pathway associated with *SREBF1*. Annotation of this gene includes the DNA and chromatin binding transcription factor activity, and it regulates the rate of transcription of the *LDL receptor* gene, fatty acid, and the cholesterol synthesis pathway to a lesser extent (Stachowiak et al., 2013).

*HMGCR* or *3-hydroxy-3-methylglutaryl coenzyme A reductase* was downregulated in the lipolysis process because the *HMGCR* gene is a protein-encoding gene; the Terpenoid backbone biosynthesis pathway is a pathway associated with this gene (Wang et al., 2018).

*Fatty acid desaturase 2* gene was upregulated in the lipogenesis process because the *FADS2* gene is a protein-encoding gene with pathways such as fatty acid beta-oxidation (peroxisome) and alpha-linolenic acid metabolism. This gene is a part of the lipid metabolic pathway that catalyzes the biosynthesis of unsaturated fatty acids from unsaturated fatty acids of linoleic acid (18:2n-6) and linolenic acid (18:3n-3) (Chen et al., 2019).

The *Stearoyl-coenzyme a desaturase* (*SCD*) gene was upregulated in the lipogenesis process, as this gene encodes the enzyme that is involved in the biosynthesis of fatty acids, so that it is first responsible for the synthesis of oleic acid. The produced protein belongs to the desaturase fatty acid family (Calvo et al., 2019).

*Acetyl-Coenzyme A acetyltransferase 1 (ACAT1)* was downregulated in the lipolysis process. This gene is a protein-encoding gene that is involved in metabolic pathways of ketone body metabolism and the Terpenoid backbone biosynthesis. The gene plays a key role in the ketone body metabolism (Chanyshev et al., 2018).

The *FASN* (*Fatty acid synthase*) gene was upregulated in the lipogenesis process because this gene is a protein-encoding gene with pathways such as the metabolism of water-soluble vitamins and cofactors, as well as the enzymatic complex pathway of AMPK. Therefore, upregulation of this gene is necessary for lipid biosynthesis (Raza et al., 2018).

The *Hydroxyacyl-CoA Dehydrogenase Trifunctional Multienzyme Complex Subunit Beta* gene was downregulated in the lipolysis process. The *HADHB* gene is a protein-encoding gene with pathways such as beta-oxidation of mitochondrial fatty acids and biosynthesis of glycerophospholipids (Diebold et al., 2019).

*Enoyl-CoA Hydratase And 3-Hydroxyacyl CoA Dehydrogenase* genes were downregulated in the lipolysis process. The *EHHADH* gene is a protein-encoding gene with pathways such as PPAR alpha pathway and propanoate metabolism. The gene annotation includes the binding of signaling receptors and oxidoreductase activity (Assmann et al., 2016). Given the ontology expression and functions of important and main genes in the network of genes interactions, it can be stated that these genes are the main genes in the metabolism and fat storage as well as the signaling pathways of endocrine glands, especially AMPK and PPAR signaling pathways.

In Figure 3, green quadrilateral nodes represent the genes with the highest interaction in the network and are the main candidates in lipid metabolism and storage. These nodes play roles in the list of desired genes (reference), metabolic, and signaling pathways. The genes with the highest repression levels include *THBS1*, *SIK1*, *COLEC12*, and *BACE1*, respectively.

A combined biological system approach is used to detect metabolic and signaling pathways associated with the interactive bipartite network of gene–miRNA in the process of fat storage and metabolism of broiler chicken. Fat stored in the skeletal muscles plays a role in important metabolic processes such as immune function, food consumption, hormone sensitivity, and relevant signaling pathways (Jung and Choi, 2014).

In module 1, *gga-miR-1710* suppressed the *HMGCR* gene and *gga-miR-1710* was downregulated. Its target gene represents the increased expression in higher abdominal fat tissue compared with lower abdominal fat tissue. The gene is classified into a set of genes associated with the lipolysis process. Reducing the expression of *gga-miR-1710* and increased gene expression of *HMGCR* leads to the lipolysis process, thereby reducing abdominal fat. *HMG-CoA reductase* protein-encoding gene is the cholesterol synthesis-limiting enzyme that regulates the product of catalyzed reactions by reductase through a negative feedback mechanism caused by sterols and non-sterol metabolites derived from mevalonate. The enzyme in mammalian cells is usually suppressed by cholesterol derived from the construction and destruction of low-density lipoprotein (LDL) through the LDL receptor (Wang et al., 2018).

The *SCD* gene indicates a higher expression in larger abdominal fat tissue compared with the lower abdominal fat tissue. The *SCD* gene is put into the set of genes associated with the lipogenesis process. Therefore, increasing the *SCD* gene expression raises the amount of fat storage in the body, especially in abdominal part. *SCD* gene (*Stearoyl-coenzyme A desaturase*) is a protein-encoding gene with pathways including adipogenesis and angiopoietin, such as the protein 8 regulatory pathway. It also plays an important role in lipid biosynthesis and regulating the expression of genes in the mitochondrial fatty acid oxidation and lipogenesis cycle (Aali et al., 2016).

*gga-miR-6554-5p* suppresses the *IRS1* gene. This miRNA has higher expression; and its target gene shows a lower expression in greater abdominal fat tissue compared with the lower abdominal fat tissue. *IRS1* gene is among the set of genes associated with the lipolysis process. Therefore, increasing expression of *gga-miR-6554-5p* miRNA decreases the *IRS1* gene expression, thereby reducing the amount of fat catabolism and increasing the abdominal fat storage and anabolism. *IRS1* gene encodes a protein that is phosphorylated by insulin receptor tyrosine kinase. Mutations in the gene are associated with type 2 diabetes and insulin resistance (Song et al., 2019).

The *SREBF1* gene shows the higher expression in greater abdominal fat tissue compared with lower abdominal fat tissue. The gene is among the set of genes associated with the lipogenesis process. The higher *SREBF1* gene expression increases the abdominal fat storage and anabolism. *SREBF1* genes encode the Helix-Loop-Helix-Leucine Zipper (bHLH-Zip) that binds the sterol-1 regulator. It is also found in the promoter for low-density lipoprotein receptor gene and other genes in the sterol biosynthesis (Stachowiak et al., 2013).

In this module, the *HMGCR* gene is suppressed by miRNAs. The gene is associated with the lipolysis process. Therefore, its suppression can prevent the fat tissue catabolism and lead to the higher fat storage and anabolism in abdominal fat tissue of broiler chickens. In this module, there are six genes, namely, *HMGCR*, *SREBF1*, *SCD*, *FASN*, *HADHB*, and *ACAT1* with certain color (green), and have the highest interaction with other genes involved in the module. The enzyme that is encoded by the *FASN* gene is a multi-functional protein. Its main function is the canalization of the synthesis of Palmitate from Acetyl*-*CoA and Malonyl-CoA in the presence of NADPH to long-chain saturated fatty acids. The *ACAT1* gene encodes a topical mitochondrial enzyme that catalyzes the reversible form of Acetoacetyl CoA from two acetyl CoA molecules. Further, the *HADHB* gene is responsible for encoding the beta subunit of mitochondrial function protein and catalyzes the final three stages of the mitochondrial beta-oxidation process of long-chain fatty acids (Diebold et al., 2019).

The gene set of this module, as presented in Table 3, encodes signaling pathways AMPK and PPAR as well as metabolic pathways of fatty acid synthase, unsaturated fatty acid synthase, and cholesterol metabolism pathways. Therefore, it can be concluded that the module and genes involved in the process can be functional modules associated with abdominal fat metabolism and storage in broiler chickens.

The receptor increases the insulin-mediated glucose uptake and improves the blood lipid profile by regulating lipid metabolism, glucose, and free fatty acid oxidation. Target genes of PPARs are related to several proteins that are necessary for absorption, intercellular transfer, and beta-oxidation of fatty acids. They include fatty acid transport proteins, the Fatty Acid Translocase enzyme, and the synthase enzyme involved in the production of acetyl CoA (for long-chain fatty acids) and Carnitine palmitoyltransferase I (Brown and Plutzky, 2007). PPARs play roles in the regulation of the gene transcription process (P2) of fat cells, so that the lean and fat-free meat can be produced by manipulation of the differentiation of fat tissue cells and their fat content through these receptors.

The cellular response to insulin includes the regulation of blood sugar levels by increasing the glucose uptake in muscles and fat tissues in a way that energy is reserved in fat tissue, liver, and muscle increase by stimulating lipogenesis, glycogen synthesis, and protein synthesis. Insulin signaling pathways decrease glucose production by the liver and the total inhibition of energy stored through lipolysis, glycogenolysis, and breakdown of proteins. This pathway also acts as a growth factor and stimulates cell growth, differentiation, and survival (Boucher et al., 2014). The insulin signaling pathway is an important biochemical pathway that regulates some basic biological functions such as glucose and lipid metabolism, synthesis of proteins, cell proliferation and differentiation, and apoptosis (Di Camillo et al., 2016).

The signaling pathway of phosphatidylinositol (PI3K)/protein kinase B (Act) is involved in the regulation of many physiological cell processes by activating effective cross-downstream molecules that play important roles in the cellular cycle, growth, and proliferation (Shi et al., 2019).

The Mammalian Target of Rapamycin (mTOR) signaling pathway has both internal and external signals and acts as a main regulator of cellular metabolism, growth, proliferation, and survival. Exploration carried out over the past decade indicates that the mTOR signaling pathway is activated in various cellular processes such as tumor formation and angiogenesis, insulin resistance, lipid metabolism, and lymphocyte T activation and is regulated in human diseases such as cancer and type 2 diabetes (Laplante and Sabatini, 2009).

In module 2, *APP* gene plays the main role. The gene is suppressed by *gga-miR-6554-5p*. *gga-miR-6554-5p* represents the upregulation; and its target gene represents the downregulation in greater abdominal fat tissue compared with the lower abdominal fat tissue. The *APP* gene is a set of genes associated with the lipolysis process. Therefore, its repression by miRNAs in humans is necessary. In poultry, its lower expression is equivalent to a decrease in abdominal fat; and a decrease in body fat is equivalent to an increase in proliferation performance and other functional traits. Increased body weight or obesity caused by increased body fat storage is characterized by excessive accumulation of fat in the body and increased levels of adipokines and inflammatory cytokines. This indicates an increased risk of Alzheimer’s disease, type 2 diabetes, and cardiovascular diseases. It has been recently found that the gene expression level of *APP* increases as brain tissue fat and fat storage tissues increase in the body (Puig et al., 2017).

*gga-miR-6554-5p* and *gga-miR-466* miRNAs suppress *BACE1* gene. These two miRNAs represent the upregulation, and their target genes indicate the downregulation in greater abdominal fat tissue compared with lower abdominal fat tissue. *BACE1* gene encodes an enzyme that cuts the amyloid precursor protein (APP) and produces amyloid beta peptides that cause amyloid plaque in the brains of patients with Alzheimer’s disease (Faghihi et al., 2008; Ghafouri et al., 2018).

*gga-miR-6562-5p* and *gga-miR-3532-5p* suppress the *PSEN1* gene. These two miRNAs indicate the upregulation; and their target gene indicates the downregulation in the greater abdominal fat tissue compared with the lower abdominal fat tissue. *PSEN1* encodes a protein that is called Presenilin 1. Presenilins are *APP* regulators according to their effects on gamma secretase as APP-decomposing enzymes (Ramakrishnan et al., 2017).

*PSEN2* gene, which has about 67% of similarity to *PSEN1* gene, was identified after *PSEN1* gene. *PSEN2* gene indicated a lower expression in greater abdominal fat tissue compared with the lower abdominal fat tissue. *PSEN2* gene is a protein-encoding gene with associated diseases such as Alzheimer’s disease and heart muscle diseases. It encodes the intermediate signaling Presenilin and Wnt/Hedgehog/Notch pathways (Muchnik et al., 2015).

*gga-miR-3532-3p* suppresses *BACE2* gene. The miRNAs indicate the upregulation; and their target gene, *BACE2*, indicates the low expression in greater abdominal fat tissue compared with lower abdominal fat tissue. *BACE2* gene encodes a full membrane glycoprotein that is known as an aspartic protease (Yu and Jia, 2009).

In module 3, the *MVD* gene indicated a higher expression in the greater abdominal fat tissue compared with the lower abdominal fat tissue. The *MVD* gene is a set of genes associated with the lipogenesis process. This gene encodes a mevalonate diphosphate decarboxylase (MVD) enzyme. Its related pathways include the protein metabolism and synthesis of available substrates in the biosynthesis of N-glycans. The *DHCR7* gene is another important gene of this module, indicating the higher expression in the greater abdominal fat tissue compared with the lower abdominal fat tissue. *DHCR7* or 7-dehydrocholesterol reductase is a protein-encoding gene that plays a role in eliminating an enzyme that creates a double bond of C (7–8) in loop B of sterol and catalyzes the conversion of 7-dehydrocholesterol to cholesterol. Cholesterol I biosynthesis and vitamin D metabolism are its associated pathways. The *TM7SF2* gene is an important paralog of this gene [see text footnote 6; 64].

Another important gene in this module, *ACAT1* gene, indicates low expression in greater abdominal fat tissue compared with the lower abdominal fat tissue. This gene catalyzes Acetoacetyl CoA using two acetyl coenzyme A molecules (Chanyshev et al., 2018).

Given the roles of the three main genes involved in the structure of this module as well as using the online database, this module encodes metabolic pathways of cholesterol metabolism and the metabolism of fatty acids.

In the Notch signaling pathway, the Notch receptor is phosphorylated and activates the *NICD* gene in collaboration with the *PSEN1* gene as a γ-secretase complex. Inside the cell nucleus, this gene encodes the sequence of the *FABP7* gene and triggers the construction of FABP7 mRNA by cooperation with RBPJ/CBF1 complex. *FABP* gene is activated by two phosphorylated receptors, called *FATP* and *FATCDB6*, in the cell membrane. Thereafter, three signaling complexes, *PPAR_α_–RXR*, *PPAR_β_–RXR*, and *PPAR_γ_–RXR*, are activated. These signaling pathways encode genes related to the fat storage and metabolism in the cell nucleus. These complexes in the nucleus are related to lipid transport, lipogenesis, cholesterol metabolism, and fatty acid oxidation, leading to the process of lipid metabolism by transcription and translation of the genes. In the signaling path of PPAR, *PPAR*_γ_/*RXR* complex is associated with the insulin-related signaling pathway through the phosphorylated mTORC1 gene in the mTOR pathway. The phosphorylation of this gene results in activation of *PPAR*_γ_/*RXR* complex. The AMPK signaling pathway is also associated with the *mTORC1* gene and has an inhibitory effect, in a way that the AMPK pathway prevents the phosphorylation of the *mTORC1* gene, so that *PPAR*_γ_/*RXR* complex is not activated; and the lipid metabolism process (e.g., lipogenesis, cholesterol, and oxidation metabolisms) is not performed. Two signaling pathways, PPAR (the main pathway of lipid metabolism) and AMPK (the main pathway of cellular energy exchanges), are important in this metabolic-signaling network. These two signaling pathways control each other by the *mTORC1* gene in the mTOR signaling path, so that increasing or decreasing the intracellular energy levels of the AMPK signaling pathway with an inhibitory or activating effect on the mTORC1 gene can cause anabolism or catabolism of lipids in cells (Figure 7).

According to the ontology and functions of genes, which encode two signaling pathways, AMPK and PPAR, these two pathways are the main pathways of cellular energy exchange and lipid metabolism, respectively.

Peroxisome proliferator-activated receptors are transcription factors belonging to the nuclear receptor superfamily, and they are activated by long-chain unsaturated fatty acids with several double bonds, eicosanoids, and lipid-lowering agents such as fibrates. Among the unsaturated fatty acids with double bonds, eicosapentaenoic acid (EPA) and docosahexaenoic acid have been widely studied because of their ability to activate PPARs. The expression profile of *PPAR*_α_ in different organs of poultry is largely similar to that of mammals, in such a way that it expresses similar functions of *PPAR*_α_in poultry and mammals. PPARs are nuclear hormone receptors that are activated by fatty acids and their derivatives. Each of them is encoded in a separate gene and bind fatty acids and eicosanoids. Ligand property of PPAR–RXR heterodimers for fatty acids causes the binding of these heterodimers to “Specific Receptor Elements” in the promoter region of several genes and changes the transcription of downstream genes involved in immune processes, lipid metabolism, and cholesterol metabolism (Zoete et al., 2007).

AMP-activated protein kinase (AMPK) is a serine/threonine kinase that has a high protective system. The AMPK system acts as a cellular energy sensor. When AMPK is activated, it simultaneously inhibits the energy consumption in biosynthetic pathways, such as protein, fatty acids, and glycogen synthesis, and activates the catabolic pathways (breakdown) of ATP production, including fatty acid oxidation and glycolysis (Miyamoto et al., 2012). The reduced regulation of liver AMPK activity plays a pathophysiological role in lipid metabolic disorders. However, the signaling pathway of AMPK for regulation of cellular energy balance is essential for the lipid metabolism, so that the pathway activates the catabolism of fat in the shortage of energy in the cell to provide the necessary rate of ATP. Therefore, the AMPK is a main regulator of cell metabolism and metabolism organ in eukaryotes, and it is activated by lowering the intra-cellular ATP level. AMPK plays an important role in the growth regulation and re-planning of cell metabolism (Mihaylova and Shaw, 2011).

## Conclusion

The combination of omics data for obtaining and identifying genes with differences in gene expression led to the successful identification of 41 genes in the main process of metabolism (anabolism and catabolism), fat storage, signaling pathways of endocrine glands, and the cell membrane in abdominal fat tissue for two groups of broiler chickens with higher and lower abdominal fat storage. The same identified genes were involved in the signaling pathways of endocrine glands; AMPK and PPAR are associated with lipid metabolism and energy catabolism and could be considered as the genes that were similar in different species. The present study identified important common genes relating to lipid metabolism and metabolic and signaling pathways, and detected mechanisms associated with lipid transfer by different cell membranes and tissues by an explanation of relevant genes. Furthermore, the gene–gene and gene–miRNA interactions were also examined by investigating the biological system and reconstruction of various regulatory and interactive networks that can affect the regulation of fat metabolism and storage in poultry. They also facilitate better understanding biology of metabolism and fat storage and the discovery of potential molecular markers in poultry industry programs to increase animal protein production efficiency and reduce abdominal fat storage.

## Data Availability Statement

All sequencing data have been submitted to the National Center for Biotechnology Information (NCBI) Gene Expression Omnibus (GEO). Each dataset contains microRNA expression raw data files (fastq format), processed data files (raw counts of sequencing reads), and a metadata spreadsheet referring to the information about the overall study and individual samples. All data can be used without restrictions. Related accession number is GSE122224. As well as reconstructed metabolic signaling pathways (in.xml format) have been submitted to the BioModels database in the European Bioinformatics Institute (EMBL-EBI). Related biomodel accession number is MODEL2010270002 (https://www.ebi.ac.uk/biomodels/).

## Ethics Statement

The animal study was reviewed and approved by The Committee on the Care and Use of Chicken Breeding Station of Tehran University farm has approved the experiments.

## Author Contributions

AB, SM-A, and MS contributed to conceptualization. AB contributed to methodology. FG and AB contributed to formal analysis. FG, SM-A, MS, and AB contributed to investigation. FG, AB, and MB contributed to data curation. FG, MB, and AB contributed to writing – original draft preparation. MB, HB, and SL contributed to writing – review and editing. MS, AB, and SM-A contributed to supervision. All authors have read and agreed to the published version of the manuscript.

## Conflict of Interest

The authors declare that the research was conducted in the absence of any commercial or financial relationships that could be construed as a potential conflict of interest.

## Footnotes

1
www.ncbi.nlm.nih.gov/geo

2
www.Ebi.ac.Uk/arrayexpress

3
http://ftp.ensembl.org/pub/release-102/fasta/gallus_gallus/dna/

4
https://david.ncifcrf.gov/

5
https://biit.cs.ut.ee/gprofiler/gost

6
https://www.genecards.org/

7
http://mirdb.org/

8
https://mirnablog.com/microrna-target-prediction-tools/

9
https://pictar.mdc-berlin.de/

10
https://tools4mirs.org/software/target_prediction/pita/

11
http://binddb.org

12
https://www.uniprot.org/database/DB-0016

13
https://thebiogrid.org/

14
http://mips.helmholtz-muenchen.de/proj/ppi/

15
https://string-db.org/

16
https://genemania.org/
