# Corrigendum: Omics multi-layers networks provide novel mechanistic and functional insights into fat storage and lipid metabolism in poultry

**DOI:** 10.3389/fgene.2025.1572670

**Published:** 2025-03-06

**Authors:** Farzad Ghafouri, Abolfazl Bahrami, Mostafa Sadeghi, Seyed Reza Miraei-Ashtiani, Maryam Bakherad, Herman W. Barkema, Samantha Larose

**Affiliations:** ^1^ Department of Animal Science, College of Agriculture and Natural Resources, University of Tehran, Karaj, Iran; ^2^ Nuclear Agriculture Research School, Nuclear Science and Technology Research Institute, Karaj, Iran; ^3^ Department of Cell and Molecular Biology, Faculty of Biological Sciences, Kharazmi University, Tehran, Iran; ^4^ Department of Production Animal Health, University of Calgary, Calgary, AB, Canada; ^5^ One Health at UCalgary, University of Calgary, Calgary, AB, Canada

**Keywords:** lipid metabolism, transcriptome, systems biology, interactive bipartite network, omics multilayer

In the published article, there was an error in **Affiliation 2**. Instead of “Biomedical Center for Systems Biology Science Munich, Ludwig-Maximilians-University, Munich, Germany,” it should be “Nuclear Agriculture Research School, Nuclear Science and Technology Research Institute, Karaj, Iran.”

The authors apologize for this error and state that this does not change the scientific conclusions of the article in any way. The original article has been updated.

