# Supplementary material for: Omics Multi-Layers Networks Provide Novel Mechanistic and Functional Insights Into Fat Storage and Lipid Metabolism in Poultry
Source: Front Genet. 2021 Jul 7;12:646297. doi: 10.3389/fgene.2021.646297 (PMC8292821; doi:10.3389/fgene.2021.646297)
Supplement: Supplementary file 1 [file Table_1.DOCX]

| **Data Type** | **Software or Package** | **Version** | **Parameters** |
| --- | --- | --- | --- |
| Microarray Datasets | Software R | R 3.5.1 | - |
|  | package Lumi | 2.42.0 | - |
|  | package Affy | 1.68.0 | - |
|  | packages Limma | 3.46.0 | - |
|  | Package GEOquary | 2.58.0 | - |
|  | Package Biobase | 2.50.0 | - |
| RNA-Seq Datasets | FastQC | Version 0.72 | length of Kmer to look for: 7 |
|  | Trimmomatic | Version 1.38.0 | Single-end reads  Number of bases to average across: 4  Average quality required: 20 |
|  | TopHat2 | Version 1.3.3 | Single-end reads  TopHat settings to use: Use defaults  Specify read group: No |
|  | CuffDiff | Version 2.2.1.6 | Library normalization method: geometric  Dispersion estimation method: Pooled  False Discovery Rate: 0.05  Min Alignment Count: 10  Perform Bias Correction: No  Include Read Group Datasets: No  Include Count Based output files: No  apply length correction: cufflinks effective length correction  Set Additional Parameters for single end reads? No  Set Advanced Cuffdiff parameters? No |

**Supplementary Table 1**. Versions and parameters were used for analysis of microarray and RNA-Seq data Sets.
